# Retraction: BRD7 Acts as a Tumor Suppressor Gene in Lung Adenocarcinoma

**DOI:** 10.1371/journal.pone.0283017

**Published:** 2023-03-29

**Authors:** The PLOS ONE Editors

The *PLOS ONE* Editors retract this article [1] because it was identified as one of a series of submissions for which we have concerns about peer review, authorship, and similarities across articles. Image similarities were noted between the 48h control panel of Fig 4D in [1] and the 72h miR-599 panel of Fig 3 in [2], and between the GAPDH panel of 3C in [1] and the GAPDH panel of Fig 5A in [3]. These concerns call into question the validity and provenance of the reported results. We regret that the issues were not addressed prior to the article’s publication.

All authors either did not reply directly or could not be reached.
